# Experimental Characterization of Inkjet-Printed Stretchable Circuits for Wearable Sensor Applications

**DOI:** 10.3390/s18103476

**Published:** 2018-10-16

**Authors:** Jumana Abu-Khalaf, Razan Saraireh, Saleh Eisa, Ala’aldeen Al-Halhouli

**Affiliations:** 1 Department of Mechatronics Engineering/NanoLab, School of Applied Technical Sciences, German Jordanian University, Amman 11180, Jordan; rsarayrah@student.aast.edu (R.S.); alaaldeen.alhalhouli@gju.edu.jo (A.A.-H.); 2 Department of Electronics & Communications Engineering, Arab Academy for Science, Technology and Maritime Transport, Cairo 11799, Egypt; saleheisa@aast.edu

**Keywords:** inkjet printing, printed sensors, silver nanoparticles, stretchable sensors

## Abstract

This paper introduces a cost-effective method for the fabrication of stretchable circuits on polydimethylsiloxane (PDMS) using inkjet printing of silver nanoparticle ink. The fabrication method, presented here, allows for the development of fully stretchable and wearable sensors. Inkjet-printed sinusoidal and horseshoe patterns are experimentally characterized in terms of the effect of their geometry on stretchability, while maintaining adequate electrical conductivity. The optimal fabricated circuit, with a horseshoe pattern at an angle of 45°, is capable of undergoing an axial stretch up to a strain of 25% with a resistance under 800 Ω. The conductivity of the circuit is fully reversible once it is returned to its pre-stretching state. The circuit could also undergo up to 3000 stretching cycles without exhibiting a significant change in its conductivity. In addition, the successful development of a novel inkjet-printed fully stretchable and wearable version of the conventional pulse oximeter is demonstrated. Finally, the resulting sensor is evaluated in comparison to its commercially available counterpart.

## 1. Introduction

Printed electronics (PE) is a technology which involves techniques developed to print electrical and electronic devices on various types of substrates. The interest in PE and its technology is growing rapidly with an expected market share of 200B USD in the next 10 years [1]. The manufacturing of conventional printed circuits employs a mix-subtractive method, while a fully additive method is employed in PE [2]. The latter method is based on the deposition of materials on the surface of a substrate and is divided into two major printing categories; contact and non-contact printing [3]. This classification is based on the presence of physical contact between the printer and the substrate. Common contact-based printing technologies include gravure printing, offset printing, flexographic printing, micro-contact printing, and offset lithography printing. Non-contact printing techniques include screen-printing, slot-die coating, and inkjet printing [4,5,6].

Photolithography techniques are widely used to fabricate electronic circuits and devices on diverse substrates (i.e., rigid, flexible, or stretchable). Recently, fabricating wearable sensors and multifunctional electronics on stretchable substrates has become a significant area of interest, due to its numerous applications. In particular, stretchable electronics are suitable for industrial, space, energy, and medical applications. In the present time, there is a high demand for stretchable electronics in the medical field. Specifically, wearable sensors can be used for monitoring vital signs of patients, such as temperature and heart rate, and for movement monitoring, such as muscle function and posture [7]. However, patterning such stretchable circuits using photolithography is costly and requires complicated fabrication steps. Specifically, conductive structures require low processing temperatures and are difficult to align. On the other hand, printing technology offers a very attractive alternative to photolithography for printed electronics [7].

Inkjet printing is considered as one of the most common printing techniques used in research. It offers a contactless method in which small drops of ink are digitally controlled; to be precisely deposited on the surface of a substrate. Moreover, it provides many appealing features such as real-time adjustment, high resolution, low cost, low processing temperature, flexible design patterns, high throughput processing, minimal usage of printing materials, less complexity of fabrication steps, compatibility with various substrates, and having no requirement for mask patterning [6,8,9,10,11,12].

Inkjet printers are classified into continuous inkjet (CIJ) printers and drop on demand (DoD) printers. In the CIJ printing mode, an electro-conductive stream of fluid is continuously delivered through a nozzle using piezoelectric actuator vibrations. DoD printers contain multiple nozzles, where the drop formation is controlled electronically. The drop jetting in DoD can be achieved either thermally by heating the cavity of the ink chamber behind the nozzle, or by actuating the membrane of the ink chamber using piezoelectric effect [13].

The ink used could be composed of metallic nanoparticles (NPs) such as silver, copper, and gold, metal–organic decomposition (MOD) ink such as β-ketocarboxylate silver, metallic nanowires (NWs) ink such as silver and copper NWs, and polymer-based conductive ink such as PEDOT/PSS. Other types of inks such as oxide ceramics and carbon nanomaterials are also possible candidates for inkjet printing [14]. Typical substrate materials used for inkjet printing are paper, glass, ceramics, polymers, textile, metal (coated with polymer or oxide layer) [13]. A key feature of inkjet printing is the printability of organic and inorganic materials, which makes it suitable for flexible, wearable, and stretchable electronics [15].

Although inkjet printing of conductive solutions has been widely investigated for microfluidic and lab-on-a-chip applications, the implementation of inkjet printing for the fabrication of stretchable circuits is still rather limited. In previous research, the main focus has been dedicated to solving problems which prevented the fabrication of conductive patterns on stretchable substrates [16,17,18]. This includes adhesion issues, and optimization of printing parameters using various types of inks and substrates. While these approaches have resulted in conductive patterns on stretchable substrates, they did not succeed in fabricating stretchable circuits; as the printed patterns could not withstand even minimal elongation. The inkjet printing approach presented in [19] has resulted in the fabrication of conductive bendable circuits on silicon rubber. However, these flexible circuits are not wearable; as they cannot sustain large stretching actions which are required when placing them on non-planar surfaces [20].

In the literature, there are two main methods which are used to fabricate stretchable circuits [21]. The first method is based on the addition of conductive fillers such as carbon nanotubes, carbon black or metallic powders to non-conductive elastomers. The second method allows the patterning of conductive lines on rubber substrates using various photolithography techniques; such as depositing conductive lines on a prefabricated wavy stretchable substrate [22], depositing conductive lines on an axially pre-stretched substrate to produce line buckling after release [23], and depositing tortuous conductive lines on a stretchable substrate [24,25,26].

In this study, we build on the latter technique by inkjet-printing sinusoidal and horseshoe conductive patterns on polydimethylsiloxane (PDMS) substrates. The wavelength, amplitude, and width of the printed traces have a major impact on the conductivity and stretchability of the printed lines along with the substrate type [16,27,28]. According to [29], PDMS is the most favorable substrate for the fabrication of wearable stretchable devices due to its flexibility, and high thermal and chemical resistance. In addition, PDMS does not cause any allergic reactions even if used for long periods of time [30].

The work presented in this paper investigates the use of inkjet printing of conductive silver nanoparticle (NP) based ink on PDMS substrates for the development of stretchable circuits, which are comparable to their photolithography-fabricated counterparts. It also introduces the design and characterization of inkjet-printed patterns based on their geometry, electrical conductivity, and critical strain. It is evident that conventional electronics are rigid, bulky, and not directly suitable for wearing. Hence, to enhance their wearability, rigid electronic circuits are either transformed into flexible and stretchable circuits or are embedded in a stretchable platform [22,31]. We have identified this as our primary objective as it can potentially create the first fully functional inkjet-printed stretchable circuit containing embedded optoelectronic components. This presents an intellectually rich set of challenges for which we propose a complete fabrication sequence. Finally, the fabricated optoelectronic sensor is evaluated in comparison to its commercially available counterpart.

## 2. Materials and Methods

Fabrication of stretchable circuits via inkjet printing mainly involves the use of conductive inks, stretchable substrates, and an inkjet printer which can produce tortuous patterns. In this section, the used materials and equipment, as well as the methodology for fabricating reliable stretchable circuits for wearable sensor applications, are introduced.

### 2.1. Materials and Equipment

#### 2.1.1. Preparation of PDMS Substrate

PDMS is a silicon-based elastomer, which is commonly used for fabricating stretchable circuits due to its durability, adjustable stiffness, biocompatibility, and commercial availability [24,26,32,33]. Dow Corning^®^ Sylgard 184, which is a transparent silicone elastomer that comes in two liquid components (base and curing agent), was chosen for this study. Both components are mixed in a 10:1 weight ratio and poured into an acrylic mold of a desired shape and thickness. Next, the mold with the liquid PDMS is degassed in a vacuum chamber for 30 min. Finally, the PDMS substrate is cured at 70 °C for 2 h in a convection oven [34,35].

Direct printing of conductive ink on PDMS is challenging due to its hydrophobic nature. In particular, the wettability of the substrate can affect the size and stability of the printed lines. Hence, it is critical to chemically or physically treat the PDMS surface prior to the inkjet printing of NP ink [34,35]. In order to improve PDMS hydrophilicity, a plasma barrel etcher is used for PDMS surface treatment (ZEPTO Diener, Ebhausen, Germany). Compared to chemical surface treatment, this method does not require the use of aggressive chemicals. Hydrophilicity of PDMS surface can be enhanced by operating the barrel etcher for oxygen plasma activation at full power (50 Watt) for 15 min.

#### 2.1.2. Inkjet Printing

In this study, silver NP based ink has been inkjet-printed on PDMS substrates (Silverjet DGP- 40LT-15C, from Sigma Aldrich, St. Louis, MO, USA). It is composed of NPs suspended in a solvent (30–35 weight % dispersion in triethylene glycol monomethyl ether). This ink has been specifically used due to its chemical stability, low chemical reactivity, and high electrical conductivity [36]. The viscosity and surface tension of sliver NP ink, as provided by the vendor, are approximately 10–18 cP and 35–40 dyn/cm, respectively. Conductive silver lines are inkjet-printed using Fujifilm Dimatix Material Printer DMP- 2831. Further details related to the printing process are mentioned in Appendix B.

#### 2.1.3. Electrical Interconnects

Non-hazardous liquid metal (EGaIn, Sunnyvale, CA, USA) has been incorporated into the fabrication of stretchable circuits [37,38,39,40,41,42,43]. Specifically, it has been used to enhance the electrical connections between the rigid electronics and the printed conductive lines [39,41] by lowering the resistance of the connection and strengthening potentially breakable points. In addition, liquid metal has been used for purposes of power transmission and signal acquisition [42,43], which allows for the development of a fully stretchable sensor with no rigid connections.

#### 2.1.4. Circuit Testing

**Figure 1:**
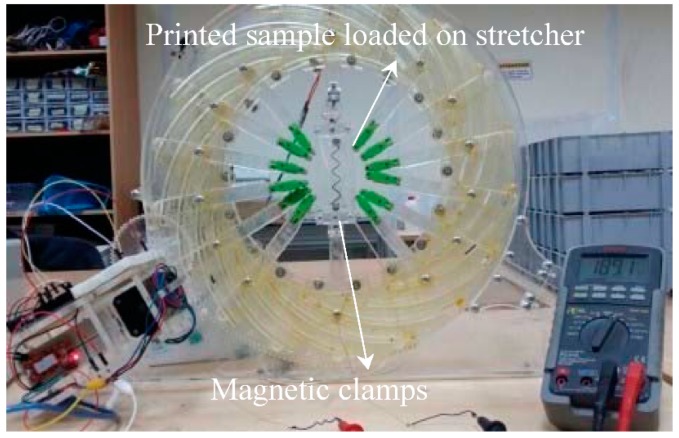
Stretching tool used to apply axial strain while measuring the electrical resistance of the printed lines.

In order to examine the stretchability of the resulting printed circuits, a custom-made automated stretching device, shown in Figure 1, is used to apply axial or radial strain on the tested samples. The used stretcher is a modified version of the stretching tool developed in [44]. The circuit under examination is held using magnetic clamps to avoid creating major stress points in the substrate. The working principle of the stretching device is based on the automatic application of axial strain on the stretchable circuit under examination, while measuring the electrical resistance of the circuit using a digital multimeter. In particular, the PDMS substrate with printed lines is extended by automatically rotating the outer frame via a stepper motor, which results in motion of the arms holding the circuit. The system is calibrated by feeding the relationship between the number of steps the stepper motor has to rotate and the applied axial strain percentage into a microcontroller. Hence, the desired strain can be entered by the user and the motor will rotate accordingly. The stretching tool calibration Arduino code is available as supplementary material.

### 2.2. Methodology

The methodology for an extensive experimental characterization of inkjet-printed stretchable circuits is presented in this subsection.

#### 2.2.1. Design of Printed Patterns

Stretchable circuits cannot be merely produced by printing straight lines on a stretchable substrate; as these lines will tend to crack once stretched. There are several fabrication techniques which allow for patterning conductive lines on a stretchable substrate and maintain conductivity while being stretched [24,26,32,33]. As previously mentioned, stretchable lines can be fabricated by depositing tortuous geometric patterns on stretchable substrates. For instance, in the work of Gray et al. [26], standard lithography was used to embed gold tortuous wires in a silicon elastomer substrate. The wires, formed of half ellipses, were optimized by varying the amplitude to wavelength ratio. Results indicated that higher amplitudes sustain higher strain when compared to lower amplitudes.

Similarly, in the approach of Brosteaux et al. [27], photolithography was used to embed a circular-based horseshoe metallic pattern in an elastic substrate. The pattern was optimized by varying the angle of the horseshoe, while fixing the amplitude to wavelength ratio. Results indicated that a horseshoe pattern with an angle of 30° is more stretchable than that with angle of 0° in the longitudinal direction. Accordingly, to be able to investigate the use of inkjet printing for the development of stretchable circuits, we propose printing sinusoidal and horseshoe conductive patterns with different properties. This will allow us to study the geometry effect on the overall circuit stretchability.

The patterns to be printed are designed using GIMP open source software, where the images are created with a desired resolution (dot per inch) then exported as a bitmap image (.bmp) and finally converted to a .ptf file by the inkjet printer software. To match the resolution of the bitmap image with the ink drop spacing (DS), we use the formula

Drop spacing (µm) = 25,400/Resolution (dpi) (1)

For example, to print patterns at DS of 30 µm, the resolution of the image needs to be 846.6 dpi.

**Figure 2:**
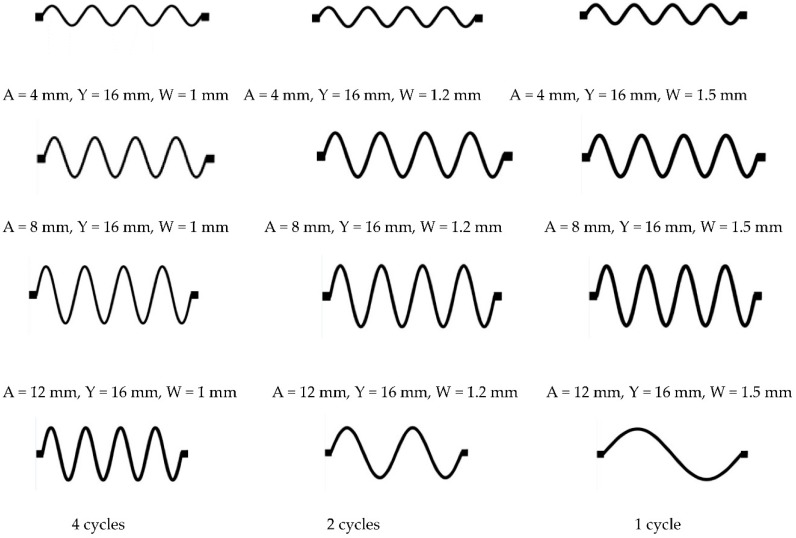
Sinusoidal patterns (not to scale) at various amplitudes, line widths, and cycles created using GIMP software.

**Figure 3:**
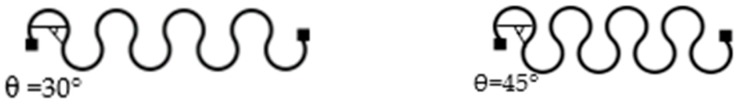
Horseshoe patterns at various angles using AutoCAD software.

To investigate the effect of the line geometry on the circuit’s stretchability, we designed sinusoidal patterns with a fixed length (L) and various line widths (W), amplitudes (A), and cycles, as illustrated in Figure 2. The number of cycles is determined by the ratio of the line length (L) to the wavelength (Y). First, we varied the amplitude and width of the lines at a constant number of cycles (four cycles). Next, we varied the number of cycles for the optimal choice of amplitude and line width found in the first step. In addition, circular-based horseshoe patterns, shown in Figure 3, were created using AutoCAD software at different angles (θ = 30° and θ = 45°), and then exported to the printer as a bitmap image using GIMP.

#### 2.2.2. Circuit Printing

**Figure 4:**
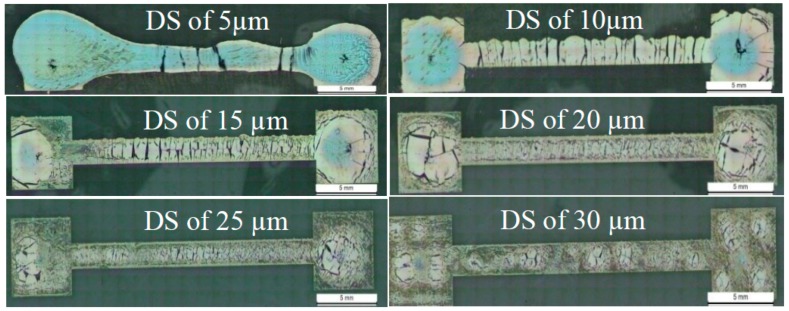
Optical microscope images of printed lines with DS of 5, 10, 15, 20, 25, and 30 µm.

There are several printing parameters which affect the conductivity, resistivity, and durability, as well as the dimensions of the printed patterns, such as drop spacing DS, cartridge/nozzle temperature, platen (substrate holder) temperature, number of printed layers, and sintering temperature [9,34]. In order to obtain uniform and continuous lines on PDMS, the printing process parameters need to be optimized. The process starts by printing simple straight lines of a width of 2000 µm with two pads at each end at different DS, which is the distance between two adjacent drops from center to center. DS highly affects the width of the printed line (as the DS increases the line width decreases) [34]. An optical microscope was used to take images of the printed lines at different DS as shown in Figure 4.

It is visually clear that increasing the DS results in uniform and continuous lines; however the resulting line width is less than that specified by the pattern image. Nevertheless, all samples were printed with the same DS so this difference can be neglected. Table 1 demonstrates the resistance and line width values corresponding to each examined DS. Based on the optical images and resistance values, all printed patterns presented in this paper were printed at DS 30 µm. The other printing parameters are set as shown in Table 2, based on recommendations introduced in [34].

**Figure 5:**
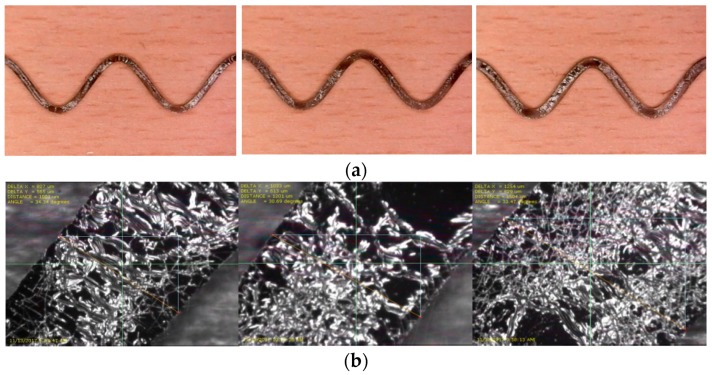
Examples of printed circuits. (**a**) Sinusoidal patterns printed at recommended parameters and widths of 1, 1.2, and 1.5 mm, respectively; (**b**) close up images of the circuits in (**a**) taken using the printer’s fiducial camera.

Examples of stretchable circuits based on the aforementioned printing parameters are shown in Figure 5. In particular, sinusoidal patterns with an amplitude of 4 mm, wavelength of 16 mm, and varying width (1, 1.2, and 1.5 mm) are printed on 3 PDMS substrates, see Figure 5a. Images taken by the printer’s fiducial camera provide a closer look at the printed patterns, see Figure 5b.

#### 2.2.3. Stretchable Sensor Development

**Figure 6:**
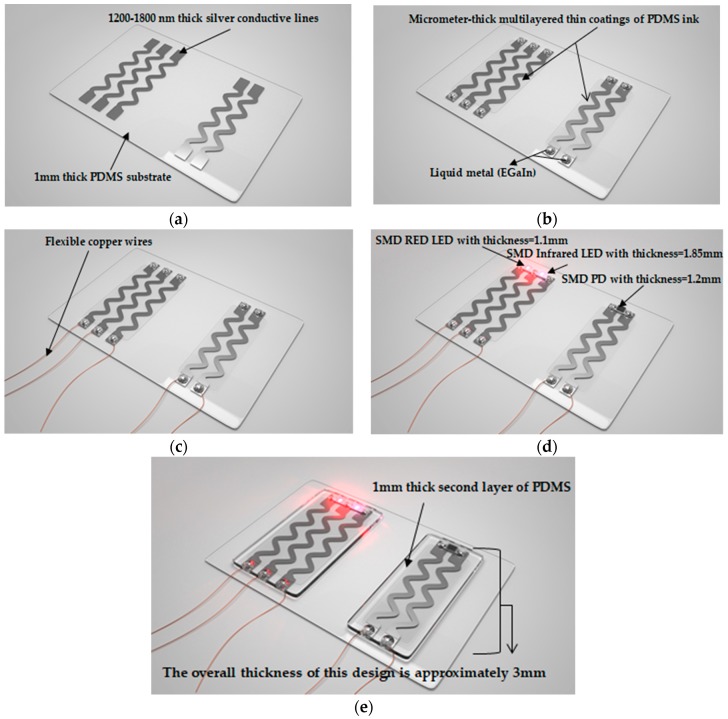
Steps for developing a fully stretchable sensor. (**a**) Sinusoidal silver conductive lines are printed on top of plasma-treated PDMS; (**b**) a protective coating of PDMS ink is inkjet-printed on top of the lines, and non-hazardous liquid metal (EGaIn, Sunnyvale, CA, USA) is incorporated for purposes of power transmission and signal acquisition; (**c**) flexible copper wires are inserted in liquid metal; (**d**) surface mount LEDs and PD are loaded on top of liquid metal; (**e**) uncured PDMS is poured to form a second layer of PDMS covering the circuit, optoelectronics, and wire connections.

After optimizing the inkjet-printed stretchable circuit, it is desired to implement the fabricated circuit into a fully stretchable sensor. This requires adding rigid electronic components to the circuit, as well as interfacing the circuit for power transmission and signal acquisition purposes. In order for the sensor to be entirely stretchable, rigid connections must be avoided. Accordingly, the steps illustrated in Figure 6 are proposed to incorporate two surface mount light emitting diodes (LEDs) and one surface mount photodiode (PD) into a stretchable platform.

In details, the process starts by printing five silver conductive sinusoidal lines, with two pads at each end, on a plasma-treated PDMS substrate, as shown in Figure 6a. Next, the printed lines are coated via inkjet printing with a thin layer of PDMS ink for protection, Figure 6b. This step is crucial to allow for covering the circuit with a second layer of PDMS. Otherwise, it was experimentally found that pouring uncured liquid PDMS directly on inkjet-printed patterns makes them nonconductive.

As liquid PDMS has a viscosity of 3900 cP, it should be diluted to be printable. The Dimatix inkjet printer requires a fluid with viscosity between 10–12 cP at jetting temperature; therefore, the mixture is diluted according to the work presented in [19]. PMDS ink can be prepared by first mixing the base with the curing agent of Sylgrad 184 in a ratio of 10:1 (base:curing agent) by weight, then diluting it with toluene 1:5 (PDMS:toluene) by volume. In this study, 15 transparent layers of PDMS ink with DS of 30 µm were inkjet-printed and successfully coated the silver conductive lines.

Subsequently, small quantities of non-hazardous liquid metal (EGaIn, Sunnyvale, CA, USA) are placed on top of the pads, Figure 6b. Flexible copper wires are inserted into the liquid metal to interface the circuit, i.e., transmit power to the LEDs and acquire the PD signal, as shown in Figure 6c. On the other end, liquid metal is used to create non-rigid low-resistance connections between the conductive lines and the surface mount optoelectronics (LEDs and PD) to be placed on top of the pads on one end, Figure 6d. Once the optoelectronics and wires are loaded in place, uncured PDMS is poured to cover the printed circuit, optoelectronics, and wire connections, as demonstrated in Figure 6e.

The overall thickness of the resulting sensor, Figure 6e, is approximately 3 mm. The sensor consists of five layers; the PDMS substrate which has a thickness of 1 mm, the printed silver conductive lines which have a thickness in the range of 1200–1800 nm [16,18], the thin coating of PDMS ink with a thickness in the micrometer range, surface mount red LED, IR LED, and PD with thicknesses of 1.1, 1.85, and 1.2 mm, respectively, and a second layer of PDMS which has a thickness of 2 mm.

## 3. Results and Discussion

In this section, experimental characterization of inkjet-printed stretchable circuits for wearable sensor applications is presented. Specifically, the optimized stretchable circuit will be implemented in a pulse oximeter sensor, which can be used to measure the human heart rate and blood oxygen saturation. Results are discussed and compared to outcomes of prominent fabrication techniques found in literature.

### 3.1. Stretchable Circuit Characterization

To begin with, sinusoidal and circular-based horseshoe patterns are inkjet-printed on PDMS with various amplitudes, line widths, cycles, and angles, as explained in the methodology section. The printed patterns will be investigated according to their ability to accommodate axial stretching while maintaining adequate electrical conductivity. The goal is to fabricate an optimal stretchable circuit, which can withstand large strain before conductivity is lost and can also be stretched for many cycles while maintaining an adequate electrical resistance.

#### 3.1.1. Effect of Conductive Line Geometry on Circuit Performance

##### Sinusoidal Patterns

**Figure 7:**
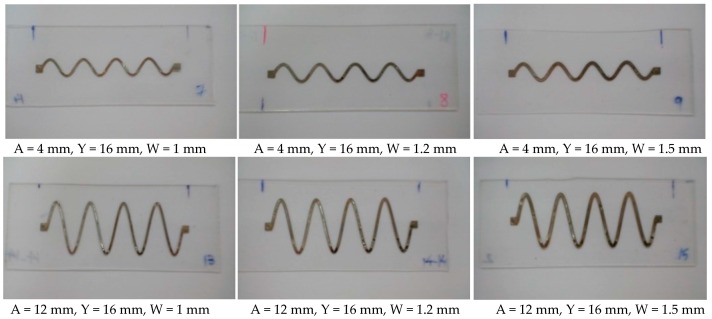
Sinusoidal conductive patterns printed on PDMS at various amplitudes and line widths.

Sinusoidal conductive patterns with different amplitude to wavelength ratios were created by varying the amplitude (A) at 4, 8, and 12 mm, while fixing the wavelength and the number of cycles at 16 mm (Y) and four cycles, respectively. The width of the lines (W) was also varied at 1, 1.2, and 1.5 mm, while maintaining a fixed length of 64 mm for all patterns. Two samples were fabricated at each line width and amplitude combination to ensure repeatability. Figure 7 demonstrates some examples of the various stretchable circuits that have been fabricated.

For each fabricated stretchable circuit, the initial line resistance (R_i_) is measured using a multimeter. Next, axial strain is applied to the circuit, using the aforementioned stretching tool, in steps of 1% up until failure; i.e., until line conductivity is lost. The resistance is measured at each step and the final resistance (R_f_), at the maximum strain before failure, is also recorded. The results are shown in Table 3, where for each circuit the initial and final resistances, as well as the maximum strain before failure are recorded.

The initial resistances were approximately in the range of a 100 Ω or less, and none of the final resistances exceeded the value of 800 Ω. It is apparent that increasing the line width and amplitude generally increased the maximum strain the line could withstand before failure. The maximum obtainable strain before failure was 21% at an amplitude of 12 mm and a line width of 1.5 mm.

**Figure 8:**
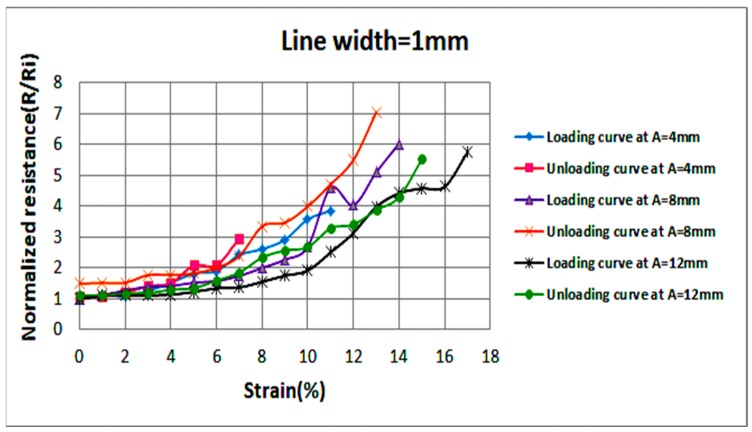
Average normalized resistances of two sinusoidal samples at a line width of 1 mm and various amplitudes as the applied strain increases (loading) and decreases (unloading).

**Figure 9:**
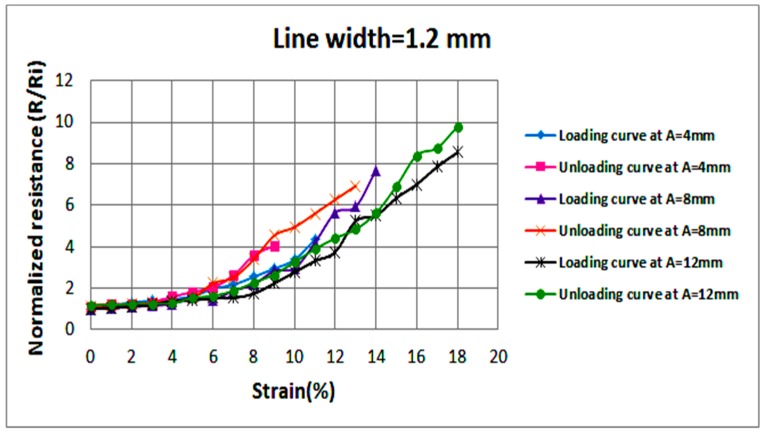
Average normalized resistances of two sinusoidal samples at a line width of 1.2 mm and various amplitudes as the applied strain increases (loading) and decreases (unloading).

**Figure 10:**
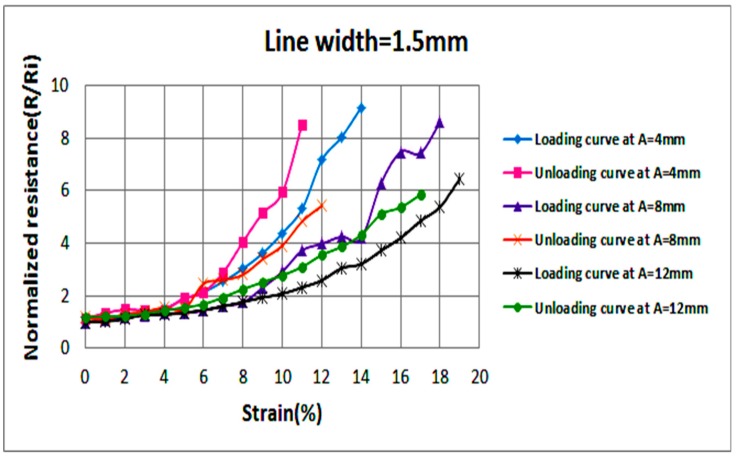
Average normalized resistances of two sinusoidal samples at a line width of 1.5 mm and various amplitudes as the applied strain increases (loading) and decreases (unloading).

**Figure A1:**
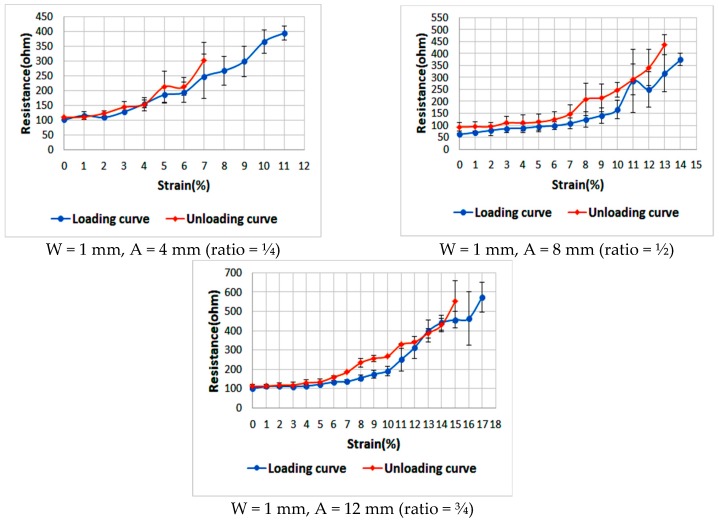
Average resistances of two sinusoidal samples at a width of 1 mm and various amplitudes as the applied strain increases (loading) and decreases (unloading). Error bars indicate the standard deviation in the values of the resistances.

**Figure A2:**
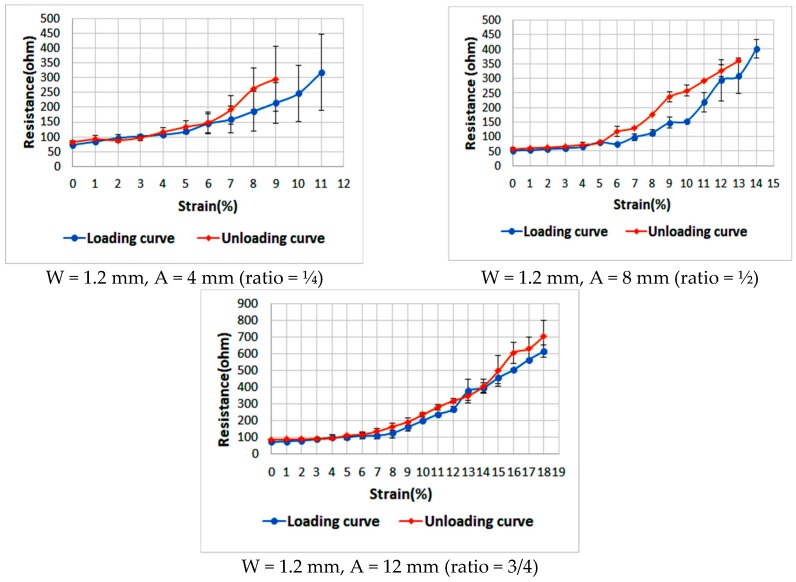
Average resistances of two sinusoidal samples at a width of 1.2 mm and various amplitudes as the applied strain increases (loading) and decreases (unloading). Error bars indicate the standard deviation in the values of the resistances.

**Figure A3:**
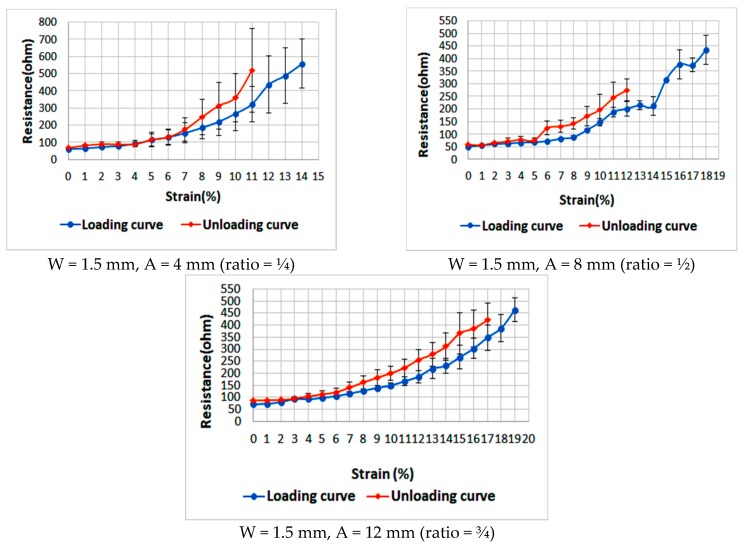
Average resistances of two sinusoidal samples at a width of 1.5 mm and various amplitudes as the applied strain increases (loading) and decreases (unloading). Error bars indicate the standard deviation in the values of the resistances.

Examining the effect of axial stretching on the stable resistance of each circuit is also crucial. Hence, once the strain at failure is reached, the circuit is released gradually by decreasing the applied strain by steps of 1% until a strain of 0% is reached; i.e., the circuit returns to its pre-stretching state. Figure 8 illustrates average normalized resistances, at each level of strain, of two samples with 1 mm wide sinusoidal lines at various amplitudes (A). Measured values of resistances are recorded as the applied strain increases (loading) and decreases (unloading). In a similar fashion, Figure 9 and Figure 10 illustrate average normalized resistances of two samples with 1.2 and 1.5 mm wide sinusoidal lines at various amplitudes. The standard deviation in the values of the resistances is indicated by error bars as shown in Figure A1, Figure A2 and Figure A3.

In the below figures, general trends show that the resistance of conductive lines increases as strain increases. Variations in sample resistance increase at larger strains, but the resistances remain within the same range of magnitude. It can also be observed that all stretchable circuits were able to return to their initial resistance as the applied strain is decreased to 0%. After reaching the strain at failure and unloading the circuit gradually, most circuits experienced loss of conductivity but became conductive again at lower strain values than those recorded during circuit loading. As can be visualized in the graphs, as the line width and amplitude increase, the circuit stretchability increases.

**Figure 11:**
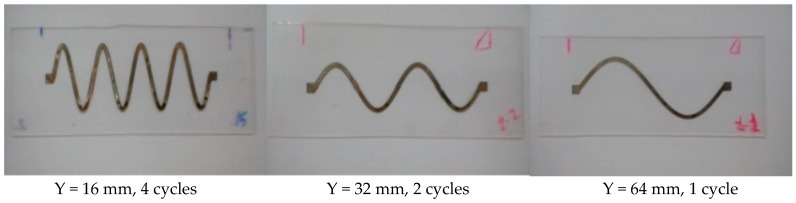
Sinusoidal conductive patterns printed on PDMS at various numbers of cycles.

**Figure 12:**
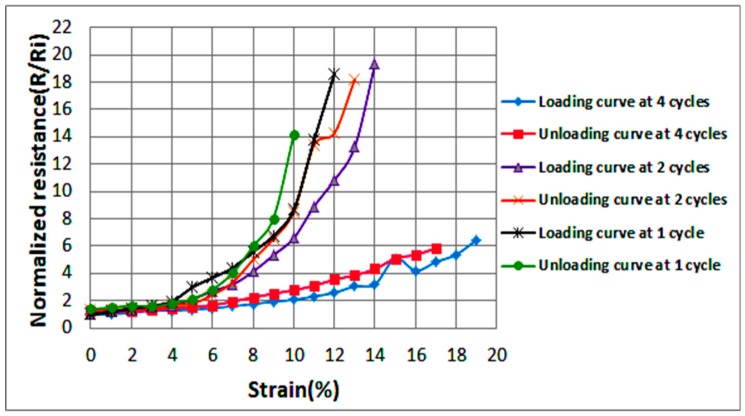
Average normalized resistances of two sinusoidal samples at various cycles as the applied strain increases (loading) and decreases (unloading).

**Figure A4:**
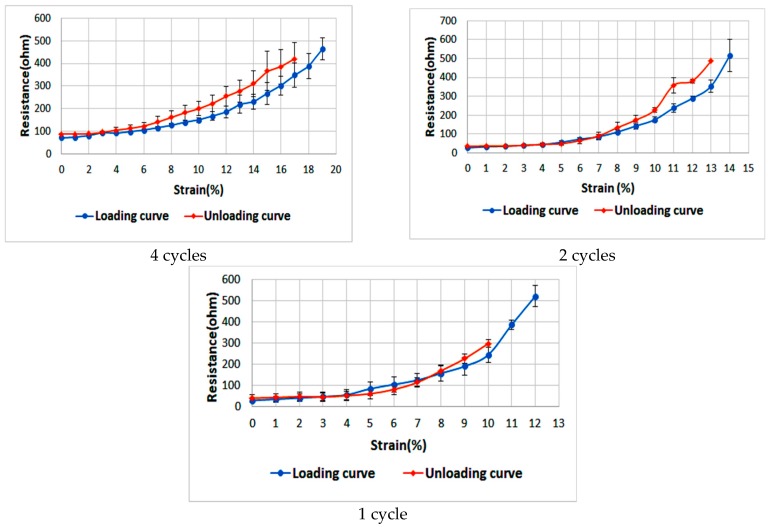
Average resistances of two sinusoidal samples at various cycles as the applied strain increases (loading) and decreases (unloading). Error bars indicate the standard deviation in the values of the resistances.

The effect of the number of cycles on the conductivity and stretchability of the printed sinusoidal circuits has also been investigated. Figure 11 demonstrates printed sinusoidal patterns at four cycles, two cycles, and one cycle. For all shown circuits the line width, amplitude, and length were fixed at 1.5, 12, and 64 mm, respectively. The number of cycles is varied by varying the wavelength (Y); since the number of cycles = length/wavelength. Figure 12 illustrates average normalized resistances of two samples at increasing strain (loading) and decreasing strain (unloading) at one, two, and four cycles. It is evident that as the number of cycles increases, the stretchability of the line increases as well. The standard deviation in the values of the resistances is indicated by error bars as shown in Figure A4.

##### Horseshoe Patterns

**Figure 13:**
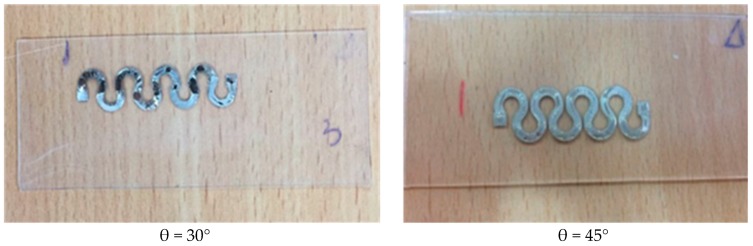
Horseshoe conductive patterns printed on PDMS at various angles.

After optimizing the parameters of sinusoidal patterns, it is desired to investigate the effect of the line shape on circuit stretchability. Accordingly, horseshoe patterns were fabricated at a line width of 1.5 mm, amplitude of 4 mm, and four cycles. The angle of the horseshoe was varied at θ = 30° and θ = 45°, as shown in Figure 13. In Table 4, the initial and final resistances, as well as the maximum strain before failure are recorded at each angle. As these patterns were created using AutoCAD the line lengths also vary as the angle changes.

**Figure 14:**
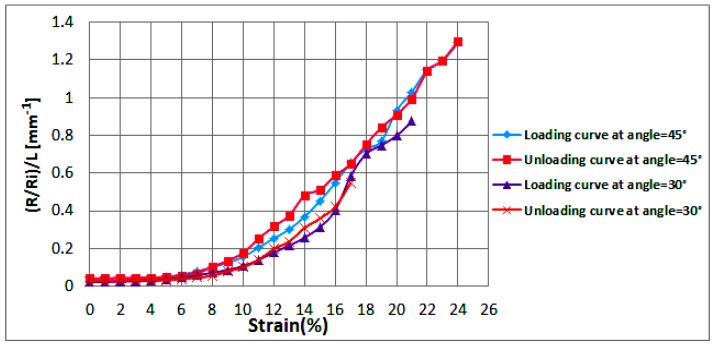
Average normalized resistances per unit length of two horseshoe samples at various angles as the applied strain increases (loading) and decreases (unloading).

**Figure A5:**
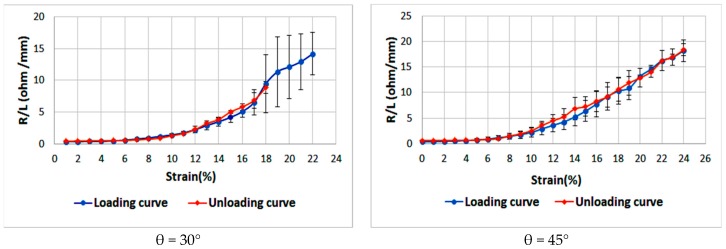
Average resistances per unit length (Ω/mm) of two horseshoe samples at various angles as the applied strain increases (loading) and decreases (unloading). Error bars indicate the standard deviation in the values of the resistances.

To be able to identify the effect of angle change on circuit stretchability, the measured resistance was divided by the line length to eliminate the influence of length. Figure 14 illustrates average normalized resistances per unit length (mm^−1^) of two horseshoe samples at increasing strain (loading) and decreasing strain (unloading) at 30° and 45°. It could be noticed that increasing the angle slightly enhanced the circuit’s stretchability from 22% to 25%. The standard deviation in the values of the resistances is indicated by error bars as shown in Figure A5.

#### 3.1.2. Evaluation of Printed Circuits

**Figure 15:**
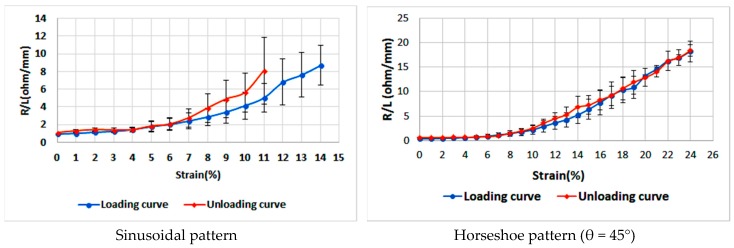
Average resistances (Ω/mm) for a sinusoidal pattern and a horseshoe pattern at A = 4 mm, W = 1.5 mm, and four cycles as the applied strain increases (loading) and decreases (unloading).

In order to compare the performance of a horseshoe pattern to a sinusoidal one, we selected samples at similar line width, amplitude, and number of cycles. Again, the measured resistance was divided by the line length to eliminate the influence of length. The data in the two graphs shown in Figure 15 was collected at an amplitude of 4 mm, a line width of 1.5, and four cycles under varying strain.

The horseshoe samples with an angle of 45° sustained an average maximum strain of 24% before losing conductivity, while the sinusoidal samples sustained an average maximum strain of 14%. The resistances for both patterns are comparable, for the sinusoidal line it was less than 10 Ω/mm, and for the horseshoe line it was less than 20 Ω/mm at maximum strain. According to the aforementioned results, an inkjet-printed horseshoe pattern with an angle of 45° is found to be a favorable choice for fabrication of stretchable circuits.

Furthermore, a cyclic strain test was applied to the optimal horseshoe sample, which sustained a maximum strain of 25%, using the aforementioned stretching tool, see Video S1. This test is used to examine the reliability of the stretchable circuit. It is desired that the circuit would undergo many cycles before failure. Accordingly, the sample was cycled up to three different values of strain: 5, 10, and 20%. Specifically, in each cycle the circuit is strained from 0% to the desired strain and then back again to 0%. At each strain, the circuit was cycled up to 3000 cycles in intervals of 500 cycles at a time.

**Figure 16:**
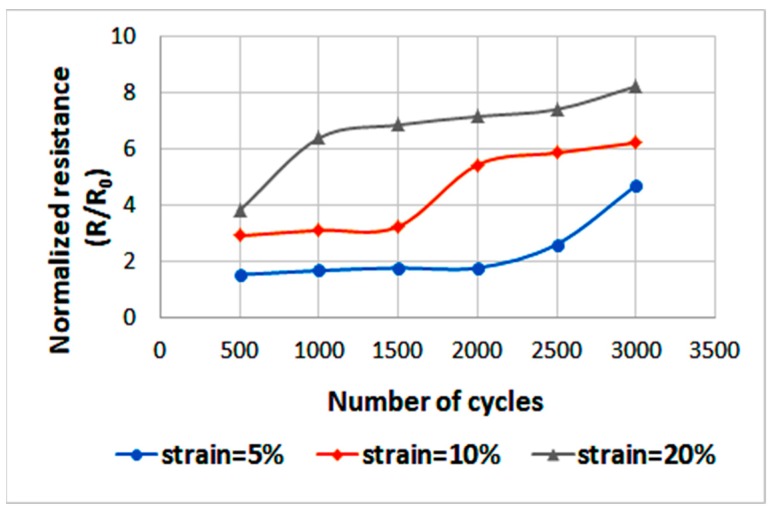
Normalized resistance (R/R_0_) for the optimal horseshoe pattern as it is cycled up to 3000 cycles at strains of 5, 10, and 20%.

The results are illustrated in Figure 16. The measured initial resistance (R_0_) is 29.5 Ω, and after completing the test and releasing the circuit, the resistance increased to 82 Ω (less than three times the initial resistance). This indicates that the stretchable circuit is indeed reliable and can be stretched for many cycles while maintaining adequate electrical conductivity.

To further assess the performance of the stretchable circuits presented in this study, we compare them to similar research endeavors found in literature. For example, silver NPs were inkjet printed on TangoBlack substrates, which are flexible and stretchable (to a certain extent) elastomers that could be printed in Objet machine [16]. Both straight lines and sinusoidal patterns of silver could not withstand the smallest amount of elongation (1 mm); however, when flexed they remained conductive up to a certain limit (up to a radius of curvature of 30 mm in the case of the sinusoidal pattern). Though these circuits were not found to be promising for stretchable electronics, they do have some potential for flexible electronics applications.

In another study [45], straight silver lines with adequate conductivity were inkjet printed on elastic thermoplastic polyurethane and silicon rubber substrates. On silicone rubber, printed lines suffered from wrinkling and cracking due to adhesion issues. Inkjet-printed silver lines on thermoplastic polyurethane had peak strains between 1.0% and 1.5% only. Nevertheless, conductivity was almost fully reversible when strain is released.

Standard lithography techniques have been used in [26] to embed both straight and tortuous gold wires PDMS. Microscopic cracks appeared when straight lines were even minimally strained (2.4 ± 0.5%). On the other hand, wires in the shape of a wave, formed of linked half-ellipses, remained conductive at much higher strain (14.2 ± 0.5%). Doubling the wave’s amplitude-to-wavelength ration improved the strain at failure to 27.2 ± 0.9%. The stretchability of the wires was found to be highly influenced by their geometry. Specifically, geometric parameters such as width, wavelength, wire type, number of wires, and depth, were examined. Narrow wires with smaller wavelength resulted in the highest strain at failure (54 ± 2%). Samples of 5 × 5 µm with amplitudes of 40 µm, and wavelengths of 80 µm were able to withstand 200 ± 30 cycles of strain. In comparison, using inkjet printing, which is a less complex and a less costly technique, we were able to achieve a circuit stretchability of up to 25% and a cycling strain of 20% up to 3000 cycles with no major effect on conductivity.

### 3.2. Wearable Sensor Application

In this section, the complete sequence required for the fabrication of a fully stretchable and wearable optoelectronic sensor, is presented. Inkjet-printed tortious patterns are implemented in a wearable version of the widely used pulse oximeter to evaluate heart (HR) and arterial oxygen saturation (SpO_2_). Pulse oximeters use optical photoplethysmography (PPG) to estimate oxygenated and de-oxygenated blood components based on their distinct light absorption coefficients. Pulse oximetry also detects photoplethysmographic (PPG) signals produced by variations in the amount of arterial blood related to the periodic contractions and relaxations of the heart [17].

Evidently, continuous monitoring of arterial oxygen saturation is important as many tissues and organs in the human body will become irreversibly damaged if not properly supplied with oxygen. However, the accuracy of pulse oximeter is significantly affected by motion artifacts; as they detect a pulsatile signal which is normally a small percentage of the total photoplethysmographic signal [46]. Hence, a wearable version of the pulse oximeter is proposed; to eliminate this limitation and lead to better sensing accuracy. Although flexible printed circuits are commonplace, they can only bend in one dimension at a time. Stretchable circuits are useful for any application where a circuit must conform to a surface with an arbitrary two-dimensional curvature, such as the human body.

Two main optical methods for oxygen saturation measurement have been developed, where the pulse oximeter operates either in the transmission or reflectance mode. In the transmission mode, the light transmitters and detector are on opposite sides of the measuring site, such that the light is transmitted through the site. While in the reflectance mode both the light transmitters and detector are mounted on the same side of the measuring site, such that the light is reflected from the measuring site back into the detector. The presented fabricated sensor is to be operated in the transmittance mode by wrapping the stretchable sensor around the human fingertip. Although horseshoe patterns performed better, they are not suitable for this application due to the dimensions of the finger area. Hence, sinusoidal patterns were implemented.

#### 3.2.1. Sensor Assembly

**Figure 17:**
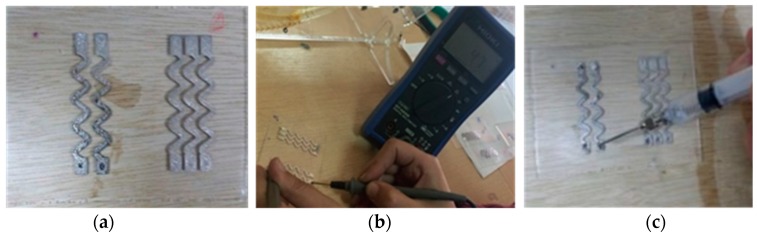

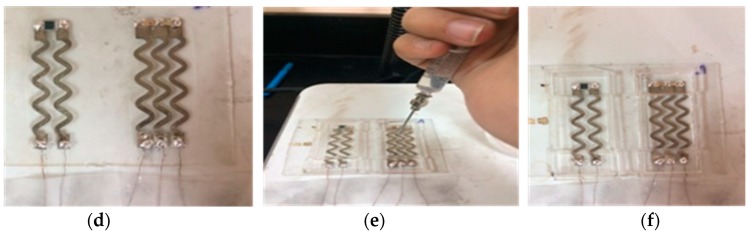
Steps for developing a fully stretchable sensor. (**a**) 2 layers of sinusoidal silver conductive lines printed on top of plasma-treated PDMS; (**b**) 15 layers of transparent PDMS ink printed to coat the silver lines, which had a resistance of 4.7 Ω; (**c**) non-hazardous liquid metal (EGaIn, Sunnyvale, CA, USA) incorporated for purposes of power transmission and signal acquisition; (**d**) surface mount LEDs and PD loaded on top of liquid metal, and flexible copper wires inserted in liquid metal; (**e**) uncured PDMS added using a syringe into a mold to form a second layer of PDMS covering the circuit, optoelectronics, and wire connections; (**f**) final sensor prototype.

As stated earlier in the methodology section, a stretchable circuit with five inkjet-printed sinusoidal lines is implemented in the development of a fully stretchable sensor, see Figure 17a. The lines have amplitude of 2 mm, wavelength of 8 mm, length of 32 mm, and a width of 1.5 mm. Next, 15 layers of transparent PDMS ink are printed to coat the silver lines and the circuit conductivity is checked, Figure 17b. Non-hazardous liquid metal is incorporated into the stretchable circuit via a syringe to control the added amount, as shown in Figure 17c. The lines are arranged such that a red LED and an infrared LED are mounted next to each other, while a PD is mounted individually, Figure 17d. This will allow for light transmittance from the LEDs to the PD through the fingertip as the sensor is wrapped around it. Afterwards, uncured PDMS is added via a syringe into a mold, which is placed on top of the circuit to control the thickness of the second PDMS layer, Figure 17e. The PDMS is cured at low temperature resulting in the final sensor prototype, Figure 17f.

**Figure 18:**
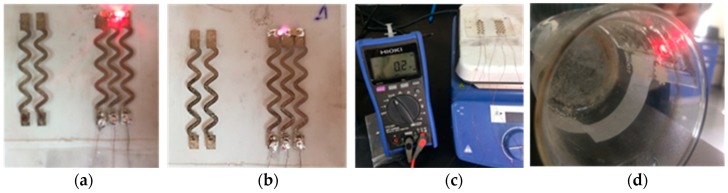
Testing stretchable sensor functionality. (**a**) Red LED is turned ON; (**b**) infrared LED is turned ON; (**c**) PD signal in response to ambient light is acquired; (**d**) red LED is ON when sensor is bent around a concave curvature.

The functionality of the optoelectronic components was tested, as shown in Figure 18. The terminals of the red LED where connected to a voltage supply and the LED illuminated, see Figure 18a. Similarly, the infrared LED was illuminated as indicated in Figure 18b. The PD diode signal at ambient light was acquired using a multimeter, as shown in Figure 18c. The sensor was functional when bent around a concave curvature, mimicking the human fingertip as indicated by the illuminated LED, Figure 18d.

#### 3.2.2. External Conditioning Circuit

An external circuitry is built to control the pulsing of the LEDs and to amplify the acquired PD signal. Also, a filtering stage is added to get rid of undesired noise. The transmitters in the pulse oximeter are composed of a red LED (660 nm) and an infrared LED (940 nm). These two LEDs are controlled by an Arduino microcontroller. Specifically, the microcontroller sends two square waves to turn the two LEDs on and off. The on and off timing should be carefully designed such that only one LED is on at a time. To control the light intensity, a resistor is added in series with each LED. The pulse oximetry Arduino code is available as supplementary material.

**Figure 19:**
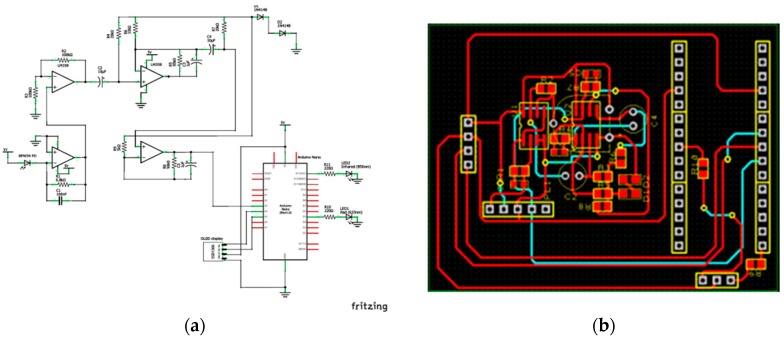
External circuitry. (**a**) Schematic diagram of external circuitry used to control the LEDs and acquire the PD signal; (**b**) PCB design of the external circuitry.

The PBW34S PD is used to convert the light signal into an electrical signal. The electrical signal’s amplitude, however, is extremely low. Thus, an amplifier is added directly after the PD to make the signal detectable. We have chosen a transimpedance amplifier (TIA) which can be used not only to amplify the signal but also to convert the PD output current into voltage. We have used the PD wizard on www.analog.com website to design a transimpedance amplifier circuit to be interfaced with the PD. The output signal from the TIA contains undesired frequency components, thus a band-pass filter is needed to remove the undesired signal and preserve the desired frequency. The schematic diagram of the external circuitry is shown in Figure 19a, and in a larger size in Figure S1. To enhance the wearability of the sensor, the circuit was built on a printed circuit board (PCB), Figure 19b.

**Figure 20:**
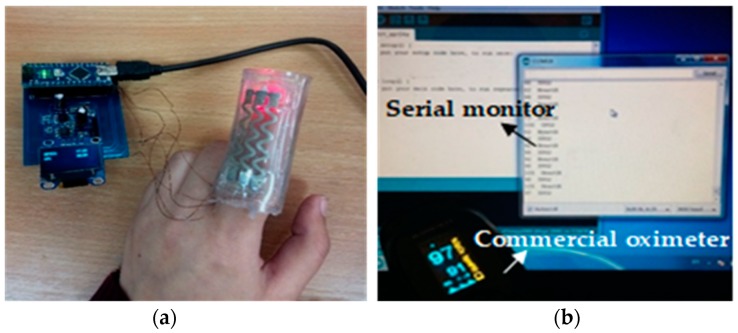
Functional stretchable pulse oximeter. (**a**) The stretchable pulse oximeter is interfaced with external circuitry; (**b**) data is collected by the sensor displayed on Arduino serial monitor and sensor output is compared to a commercial pulse oximeter.

The fully stretchable pulse oximeter is placed on the fingertip of a human subject and interfaced with the external circuitry, as shown in Figure 20a. Next, the heart rate and oxygen saturation data are displayed on the Arduino serial monitor, Figure 20b. To evaluate the performance of the sensor, its output is compared to that of a commercially available pulse oximeter (JPD-500D, Jumper, Shenzhen, China), Figure 20b. Preliminary results indicate that the stretchable and wearable version of the pulse oximeter is comparable to the commercial one. This confirms the potential of inkjet-printed stretchable circuits in wearable sensor applications.

To further examine the capabilities of the developed stretchable pulse oximeter, it was tested on a representative population (10 females and 10 males). The measured values of SpO_2_ and pulse heart rate (HR) for the 20 human subjects were compared to those measured using the commercial fingertip pulse oximeter. Results showed an average error of ±4.72% for HR and of ±0.755% for SpO_2_, see Table 5. This indicates that the developed stretchable and wearable pulse oximeter is indeed comparable to the commercial one. Additionally, the fabrication methodology presented in this paper can be applied to other optoelectronic sensors with analogous principles of operation.

## 4. Conclusions

This paper presented a very successful and complete sequence for the fabrication of a fully stretchable and wearable inkjet-printed optoelectronic sensor. The widely used pulse oximeter has been successfully fabricated in a stretchable and wearable version and evaluated in comparison to a commercially available pulse oximeter. Extensive experimental characterization for optimizing the design of the inkjet-printed conductive patterns on stretchable PDMS substrate, have been conducted. Sinusoidal and circular-based horseshoe patterns with various amplitudes, line widths, cycles, and angles were examined. The printed circuit showed its ability to accommodate axial stretching up to a strain of 25% using the horseshoe pattern at an angle of 45° while maintaining electrical conductivity with a resistance under 800 Ω. The conductivity of the circuit is fully reversible once it is returned to its pre-stretching state. It could also undergo up to 3000 stretching cycles without exhibiting a significant change in its conductivity. Obtained results indicate that the stretchable and wearable pulse oximeter is comparable to the commercial one. Moreover, the presented fabrication methodology can also be applied to optoelectronic sensors with analogous principles of operation.

## Figures and Tables

**Table 1:** Line width and resistance at different DS.

Drop Spacing (µm)	Resistance (ohm)	Line Width (µm)
5	0.4	2591.2
10	1.5	1862.6
15	2.6	1805.1
20	8	1620.2
25	15	1612
30	24.9	1611.9

**Table 2:** Printing parameters.

Printing Parameters	Values
Nozzle/cartridge temperature	32 °C
Platen temperature	26 °C (room temperature)
Firing voltage	23 volts
Speed	8 m/s
Sintering temperature and time	150 °C for 1 h

**Table 3:** Effect of sinusoidal line amplitude and width on initial and final resistances, as well as the maximum strain before failure.

Amplitude (mm)	Width (mm)					
	1		1.2		1.5	
	Sample 1	Sample 2	Sample 1	Sample 2	Sample 1	Sample 2
4 (Ratio = ¼)	R_i_ = 102.1 Ω R_f_ = 372.1 Ω S% = 11%	R_i_ = 102.8 Ω R_f_ = 417.6 Ω S% = 11%	R_i_ = 72.1 Ω R_f_ = 269.2 Ω S% = 12%	R_i_ = 74.3 Ω R_f_ = 446.2 Ω S% = 11%	R_i_ = 59 Ω R_f_ = 0.7 KΩ S% = 14%	R_i_ = 62.3 Ω R_f_ = 0.6 KΩ S% = 16%
8 (Ratio = ½)	R_i_ = 65 Ω R_f_ = 0.4 KΩ S% = 14%	R_i_ = 59.2 Ω R_f_ = 349.9 Ω S% = 15%	R_i_ = 55.5 Ω R_f_ = 389.3 Ω S% = 15%	R_i_ = 48.6 Ω R_f_ = 432.6 Ω S% = 14%	R_i_ = 53.6 Ω R_f_ = 493.3 Ω S% = 18%	R_i_ = 47.2 Ω R_f_ = 426.4 Ω S% = 19%
12 (Ratio = ¾)	R_i_ = 105.5 Ω R_f_ = 0.8 KΩ S% = 17%	R_i_ = 94.2 Ω R_f_ = 497 Ω S% = 18%	R_i_ = 72.6 Ω R_f_ = 0.65 KΩ S% = 18%	R_i_ = 71.3 Ω R_f_ = 0.56 KΩ S% = 18	R_i_ = 68 Ω R_f_ = 461.7 Ω S% = 21%	R_i_ = 75.6 Ω R_f_ = 513.6 Ω S% = 19%

**Table 4:** Effect of horseshoe angle on initial and final resistances, as well as the maximum strain before failure.

Theta = 30° L = 45 mm		Theta = 45° L = 37.8 mm	
Sample 1	Sample 2	Sample 1	Sample 2
R_i_ = 15.2 Ω R_f_ = 700 Ω S% = 24%	R_i_ = 17.2 Ω R_f_ = 790 Ω S% = 21%	R_i_ = 13.6 Ω R_f_ = 622.8 Ω S% = 25%	R_i_ = 14.7 Ω R_f_ = 770 Ω S% = 24%

**Table 5:** Experimental values measured for 20 human subjects using developed stretchable oximeter.

Subject	Gender	Heart Rate (HR)		Oxygen Saturation (SPO_2_)	
		Observed	Actual	Observed	Actual
1	Female	91	94	97	97
2	Female	78	80	98	98
3	Female	61	66	99	99
4	Female	89	81	96	97
5	Female	75	79	98	96
6	Female	72	75	98	98
7	Female	82	78	99	98
8	Female	82	81	99	97
9	Female	81	77	95	98
10	Female	86	84	98	98
11	Male	78	79	97	98
12	Male	74	71	96	96
13	Male	84	80	98	98
14	Male	80	81	97	98
15	Male	89	90	98	98
16	Male	85	91	96	96
17	Male	75	70	98	98
18	Male	79	72	95	97
19	Male	77	80	99	98
20	Male	87	80	97	95

## Supplementary Materials

The following are available online at http://www.mdpi.com/1424-8220/18/10/3476/s1, Figure S1: External circuitry for pulse oximeter, Video S1: Cyclic test, Stretching tool calibration Arduino code, Pulse oximetry Arduino code.

## Appendix B

Fujifilm Dimatix Material Printer DMP- 2831 uses piezoelectric inkjet technology, and offers a cost-effective, easy-to-use, and precise deposition system. It allows for the deposition of numerous fluidic materials on various substrates. Additionally, the system has a waveform editor and a drop-watch camera that allow for the manipulation of the electronic pulses sent to the piezo jetting device; in order to optimize the printing parameters. In addition, the temperature of the substrate holder (platen) can be adjusted up to 60 °C when needed.

Ink comes in the form of cartridges containing a reservoir with a capacity of 1.5 mL, in order to reduce the waste of expensive fluids. Cartridges can easily be replaced to facilitate printing of a series of different fluids. Each single-use cartridge has 16 nozzles linearly spaced at 254 microns with typical droplet sizes of 1 and 10 picoliters. In order to print a pattern, a desired plot is fed to the printer using a pattern editor program. This printing system enables easy printing of samples for process verification and prototype creation.
