# Supplementary material for: Experimental Characterization of Inkjet-Printed Stretchable Circuits for Wearable Sensor Applications
Source: Sensors (Basel). 2018 Oct 16;18(10):3476. doi: 10.3390/s18103476 (PMC6210026; doi:10.3390/s18103476)
Supplement: Supplementary file 1 [file sensors-18-03476-s001.zip › External circuitry for pulse oximeter.pdf]

Supplementary Materials

Experimental Characterization of Inkjet-Printed Stretchable Circuits for Wearable Sensor Applications

Jumana Abu-Khalaf [1,*], Razan Saraireh [2], Saleh Eisa [2], and Alaaldeen Al-Halhouli [1]

[1] Department of Mechatronics Engineering/NanoLab, School of Applied Technical Sciences, German Jordanian University, Amman, Jordan; jumana.abukhalaf@gju.edu.jo; alaaldeen.alhalhouli@gju.edu.jo

[2] Department of Electronics & Communications Engineering, Arab Academy for Science, Technology and Maritime Transport, Cairo, Egypt; rsarayrah@student.aast.edu; saleheisa@aaast.edu

* Correspondence: jumana.abukhalaf@gju.edu.jo; Tel.: +962-799-213713

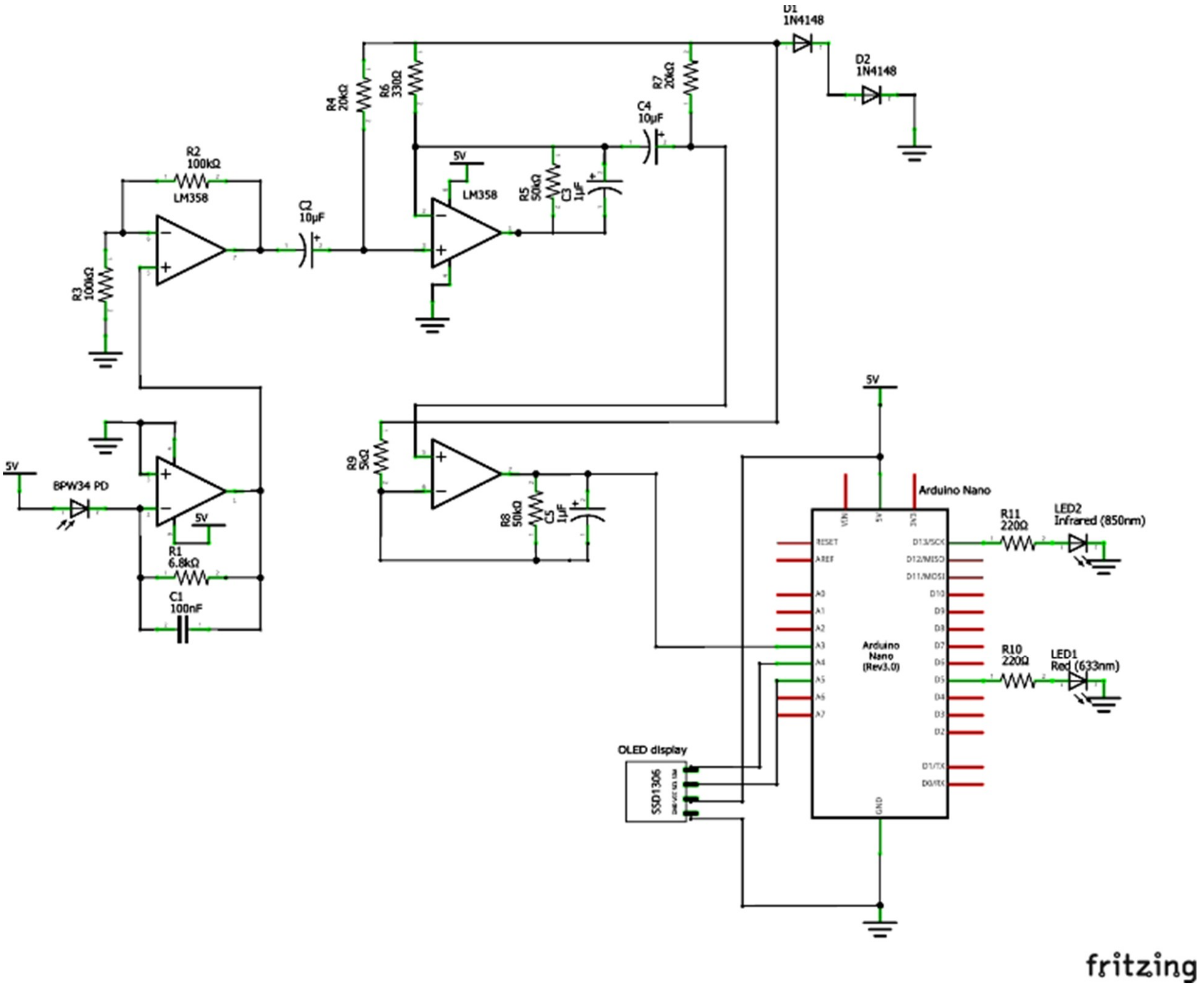

Figure S1: External circuitry for pulse oximeter
